# *Blastocystis* in free-ranging wild ruminant species across the Iberian Peninsula

**DOI:** 10.1186/s13567-025-01563-3

**Published:** 2025-07-09

**Authors:** Ana M. Figueiredo, Alejandro Dashti, Jenny G. Maloney, Aleksey Molokin, Nadja S. George, Pamela C. Köster, Begoña Bailo, Ana Sánchez de las Matas, Miguel Ángel Habela, Antonio Rivero-Juarez, Joaquín Vicente, Emmanuel Serrano, Maria C. Arnal, Daniel Fernández de Luco, Patrocinio Morrondo, José A. Armenteros, Ana Balseiro, Guillermo A. Cardona, Carlos Martínez-Carrasco, Rita T. Torres, Carlos Fonseca, Atle Mysterud, João Carvalho, Rafael Calero-Bernal, David González-Barrio, Mónica Santín, David Carmena

**Affiliations:** 1 Department of Biology and CESAM, University of Aveiro, Aveiro, Portugal; 2 Centre for Ecological and Evolutionary Synthesis, Department of Biosciences, University of Oslo, Oslo, Norway; 3 Parasitology Reference and Research Laboratory, Spanish National Centre for Microbiology, Health Institute Carlos III, Majadahonda, Madrid Spain; 4 Agricultural Research Service, United States Department of Agriculture, Environmental Microbial and Food Safety Laboratory, Beltsville, MD USA; 5 Faculty of Health Sciences, Alfonso X El Sabio University (UAX), Villanueva de La Cañada, Spain; 6 Faculty of Medicine, Alfonso X El Sabio University (UAX), Villanueva de La Cañada, Spain; 7 Department of Animal Health, Veterinary Sciences Faculty, University of Extremadura, Cáceres, Spain; 8 Infectious Diseases Unit, Maimonides Institute for Biomedical Research (IMIBIC), University Hospital Reina Sofía, University of Córdoba, Córdoba, Spain; 9 Center for Biomedical Research Network in Infectious Diseases (CIBERINFEC), Health Institute Carlos III, Madrid, Spain; 10 SaBio Group, Institute for Game and Wildlife Research, IREC (UCLM-CSIC-JCCM), Ciudad Real, Spain; 11 Wildlife Ecology & Health Group (WE&H), Wildlife Environmental Pathology Service (SEFaS), Department of Animal Medicine and Surgery, Autonomous University of Barcelona, Bellaterra, Spain; 12 Department of Animal Pathology, Veterinary Faculty, University of Zaragoza, Saragossa, Spain; 13 INVESAGA Group, Department of Animal Pathology, Faculty of Veterinary, University of Santiago de Compostela, Lugo, Spain; 14 Council of Development, Territory Planning and the Environment of the Principado de Asturias, Oviedo, Spain; 15 Animal Health Department, Veterinary School, University of León, León, Spain; 16 Animal Health Department, Mountain Livestock Institute (CSIC-University of León), León, Spain; 17 Livestock Laboratory, Regional Government of Álava, Vitoria-Gasteiz, Spain; 18 Animal Health Department, University of Murcia, Espinardo, Murcia Spain; 19 SALUVET, Department of Animal Health, Faculty of Veterinary, Complutense University of Madrid, Madrid, Spain

**Keywords:** Barbary sheep, deer, epidemiology, Iberian ibex, mouflon, NGS, Portugal, subtype diversity, Southern chamois, Spain, zoonoses, ST49

## Abstract

The growing wild ungulate populations across Europe represents an increasingly important source for the spread of zoonotic pathogens. *Blastocystis* is a common intestinal protist observed in humans and animals worldwide. Studies on *Blastocystis* occurrence and subtype (ST) diversity in free-ranging wild ruminants are lacking globally, and more data are needed to understand the epidemiological scenario in wild European ruminants. We collected 833 faecal samples from free-ranging wild ungulates across Spain (*n* = 699) and Portugal (*n* = 134) between 1998 and 2021. Using conventional PCR and next-generation amplicon sequencing, *Blastocystis* was found in 13.8% (115/833; 95% CI: 11.5–16.3) of the wild ruminants analysed. Its occurrence was significantly higher in Portugal (38.1%, 51/134; 95% CI 29.8–46.8) than in Spain (9.2%, 64/699; 95% CI: 7.1–11.5). Fifteen *Blastocystis* STs, fourteen previously recognised (ST2, ST5, ST10, ST13, ST14, ST21, ST23–ST26, ST30, and ST42–ST44), and one novel (named ST49), were detected among the surveyed wild ruminant populations. Novel ST49 was described using Oxford Nanopore sequencing to produce full-length reference sequences of the small subunit ribosomal RNA gene. A greater ST diversity was observed in Spanish samples. Mixed infections were found in 58.3% (67/115) of the total *Blastocystis*-positive samples. Our results have enhanced the knowledge regarding *Blastocystis* occurrence and ST diversity and host preference present in wild ruminants from the Iberian Peninsula, which will assist in clarifying the relationships between the sylvatic and domestic cycles of this protist and may ultimately provide tools to help manage future public health epidemiological scenarios.

**Supplementary Information:**

The online version contains supplementary material available at 10.1186/s13567-025-01563-3.

## Introduction

In recent decades, an increase in the number and distribution of wild ungulate populations has occurred across Europe, in some cases labelled as an overabundance scenario [1, 2]. This exponential population growth can be attributed to several factors, including the abandonment of land and consequential habitat re-naturalisation, local reintroductions, a reduction in native predators, and stricter hunting legislation coupled with a trending decrease in hunters’ numbers [3–5]. This situation has created the need for new ungulate management and conservation paradigms to address the negative consequences of wild ungulate overpopulation [1]. The rapid wild ungulate expansion has been associated with damage in agriculture, forestry, and woodlands, with cascading effects on species communities (e.g., invertebrates, birds, and small mammals), an increased number of ungulate-vehicle collisions, and the spread of infectious diseases [6–9]. This last issue has gained particular attention over the past few years, especially under the One Health framework, triggering new scientific debates and leading to new approaches that recognise the strong connection of emerging infectious diseases (EIDs) with the environment and human activities [10, 11]. It is estimated that nearly 60–80% of EIDs are caused by pathogens with zoonotic potential, and at least 70% have a wildlife origin [12]. However, even though both wild and domestic animals can be sources of EIDs, the ongoing anthropogenic influence on ecosystems still plays a fundamental role in managing disease risk at the wildlife-domestic-human interface [11]. Among emergent zoonotic microorganisms, *Blastocystis* is a eukaryote belonging to the Stramenopile group recognised for infecting the gastrointestinal tract of humans and animals worldwide [13, 14]. This faecal-oral transmitted protist has been linked with gastrointestinal illnesses, including abdominal pain, diarrhoea, and irritable bowel syndrome, but also urticaria [15]. Additionally, asymptomatic carriage is common, with studies indicating that *Blastocystis* can occur in the gut microbiota of healthy humans and animals [16, 17]. There is no clear evidence suggesting that *Blastocystis* has a pathogenic role in infected animals [14]. However, understanding the epidemiology of the protist in domestic and wildlife reservoirs is important to identify potential sources of infection and transmission routes to humans.

*Blastocystis* genetic heterogeneity is substantial, and, based on polymorphism in the small subunit ribosomal RNA (*ssu* rRNA) gene, up to 44 genetically distinct subtypes (STs) have been described (ST1–ST17, ST21, ST23–ST48) [18–23]. Nearly 90% of the *Blastocystis* isolates of human origin characterised worldwide have been associated with ST1-ST4 infections [24]. Ten additional subtypes (ST5–ST10, ST12, ST14, ST16, and ST23) have been reported in humans and animals [25–27] and two (ST35 and ST41) only in humans [18, 28], suggesting that some zoonotic STs have low host preference [14]. In fact, previous studies conducted on wild ungulates, primarily wild ruminants, have already described the presence of potentially zoonotic STs, such as ST4 and ST10 in red deer (*Cervus elaphus*), ST5 and ST10 in roe deer (*Capreolus capreolus*) and fallow deer (*Dama dama*), ST10 in Barbary sheep (*Ammotragus lervia*), and ST14 in mouflon (*Ovis gmelini*) (Table 1). It is worth noting that ST5, ST10, and ST14 have only been sporadically found in humans. It also should be noted that ST14 sequences reported before 2023 should be re-evaluated, as recent metagenomic and phylogenetic analyses have demonstrated that some of them may correspond to ST24 intra-subtype variants [19, 21]. However, studies reporting *Blastocystis* ST diversity in wild ruminants are still limited, and no study has been conducted thus far assessing their presence in endemic ruminant species such as the Iberian ibex (*Capra pyrenaica*) and the Southern chamois (*Rupicapra pyrenaica pyrenaica*) (Table 1). To fill this knowledge gap, this study aims to determine the occurrence, intra- and inter-ST diversity, and zoonotic potential of *Blastocystis* in the wild ruminant species inhabiting the Iberian Peninsula (Portugal and Spain) using next-generation amplicon sequencing (NGS).

**Table 1:** **Occurrence and molecular diversity of** ***Blastocystis*** **reported in the wild ruminants globally**

Host scientific name	Host common name	Country	Population status	Prevalence % (no. pos/total)	Detection method	Subtype(s) (*n*)	Mixed ST’s detected?	References
*Cervus elaphus*	Red deer	Australia	Wild	27 (19/70)	PCR, NGS	**ST10** (6), **ST14** (8), ST21 (9), **ST23** (4), ST24 (10), ST25 (3)	Yes	[22]
		Australia	Wild	2 (1/50)	PCR, SS	**ST4** (1)	No	[29]
		China	Captive	33 (1/3)	PCR, SS	**ST10** (1)	No	[30]
		China	Captive	40 (2/5)	PCR, SS	**ST10** (2)	No	[31]
		United Kingdom	Captive	100 (1/1)^a^	PCR, SS	**ST4** (2**), ST10** (6)	ND	[32]
		United Kingdom	Captive	33 (1/3)^a^	PCR, SS	**ST4** (1**), ST10** (1)	ND	[33]
		Poland	Wild	25 (2/8)	PCR, SS	**ST1** (2)	No	[34]
		Portugal	Wild	40 (38/94)	PCR, NGS	**ST2** (1), **ST5** (4), **ST10b** (5), ST21 (5), ST24a (11), ST24b (32), ST24c (13), ST25 (1)	Yes	This study
		Spain	Wild	1^**b**^	PCR, NGS	ST25 (1), ST42a (1)	Yes	[19]
		Spain	Wild	12 (39/329)	PCR, NGS	**ST5** (1), **ST10a** (6), **ST10b** (9), **ST10**^**c**^ (3), ST13 (8), **ST14** (3), ST21 (3), **ST23** (3), ST24a (26), ST24b (20), ST24c (6), ST43 (1), ST44 (3)	Yes	This study
*Capreolus capreolus*	Roe deer	Denmark	Captive	1^b^	PCR, SS	**ST10** (1)	ND	[35]
		United Kingdom	Captive	50 (1/2)	PCR, SS	**ST5** (1)	No	[36]
		Portugal	Wild	1^b^	PCR, NGS	**ST10b** (1)	ND	[19]
		Portugal	Wild	33 (13/40)	PCR, NGS	**ST5** (2), **ST10b** (1), ST13 (10), ST44 (1), ST49 (3)	Yes	This study
		Spain	Wild	13 (12/93)	PCR, NGS	**ST2** (1), **ST10a** (1), **ST10b** (2), ST13 (7), ST24a (1), ST24b (2), ST49 (2)	Yes	This study
*Dama dama*	Fallow deer	Australia	Wild	63 (31/49)	PCR, NGS	**ST10** (22), **ST14** (24), ST21 (22), **ST23** (18), ST24 (25), ST25 (19)	Yes	[22]
		China	Captive	31 (4/13)	PCR, SS	**ST5** (1), **ST10** (3)	No	[37]
		Mauritius	Captive	100 (2/2)	PCR, SS	**ST10** (2)	No	[36]
		China	Captive	50 (1/2)	PCR, SS	**ST10** (1)		[30]
		Italy	Captive	100 (1/1)	PCR, SS	**ST5** (1)	No	[38]
		Spain	Wild	4 (4/96)	PCR, NGS	**ST5** (1), **ST10a** (3), **ST10b** (1), **ST14** (4), ST21 (2), ST24a (1), ST24b (1), ST24c (1), ST25 (1), ST26 (1), ST30 (1), ST42b (2), ST43 (1), ST44 (1)	Yes	This study
*Ammotragus lervia*	Barbary sheep	China	Captive	100 (3/3)	PCR, SS	**ST5** (1), **ST10** (1), **ST14** (1)	No	[37]
		Libya	Captive	20 (1/5)	PCR, SS	**ST10** (1)	No	[36]
		Spain	Wild	10 (2/20)	PCR, NGS	**ST2** (1), ST24b (2)	Yes	This study
*Ovis gmelini*	Mouflon	Czech Republic	Captive	100 (1/1)	PCR, SS	**ST14**^**d**^ (1)	No	[36]
*Capra pyrenaica*	Iberian ibex	Spain	Wild	4 (4/89)	PCR, NGS	**ST2** (2), ST13 (3), ST24b (1)	Yes	This study
*Rupicapra pyrenaica pyrenaica*	Southern chamois	Spain	Wild	5 (3/62)	PCR, NGS	**ST5** (1), **ST10b** (2), **ST14** (1), ST21 (1), **ST23** (1), ST24a (1), ST24b (1), ST24c (1), ST44 (1)	Yes	This study

Potentially zoonotic subtypes are in bold

ND, Not determined; NGS, next-generation amplicon sequencing; PCR, Polymerase chain reaction; SS, Sanger sequencing

^a^The number of STs identified is based on the number of sequence-positive clones from the PCR-positive samples obtained in the study

^b^These studies were not planned as surveys

^c^Potential novel ST10 subgroup based on sequence similarity and phylogenetic clustering

^d^Renamed as ST24b according to the current literature. See references [19] and [21]

## Materials and methods

### Sampling sites and sample collection

**Figure 1:**
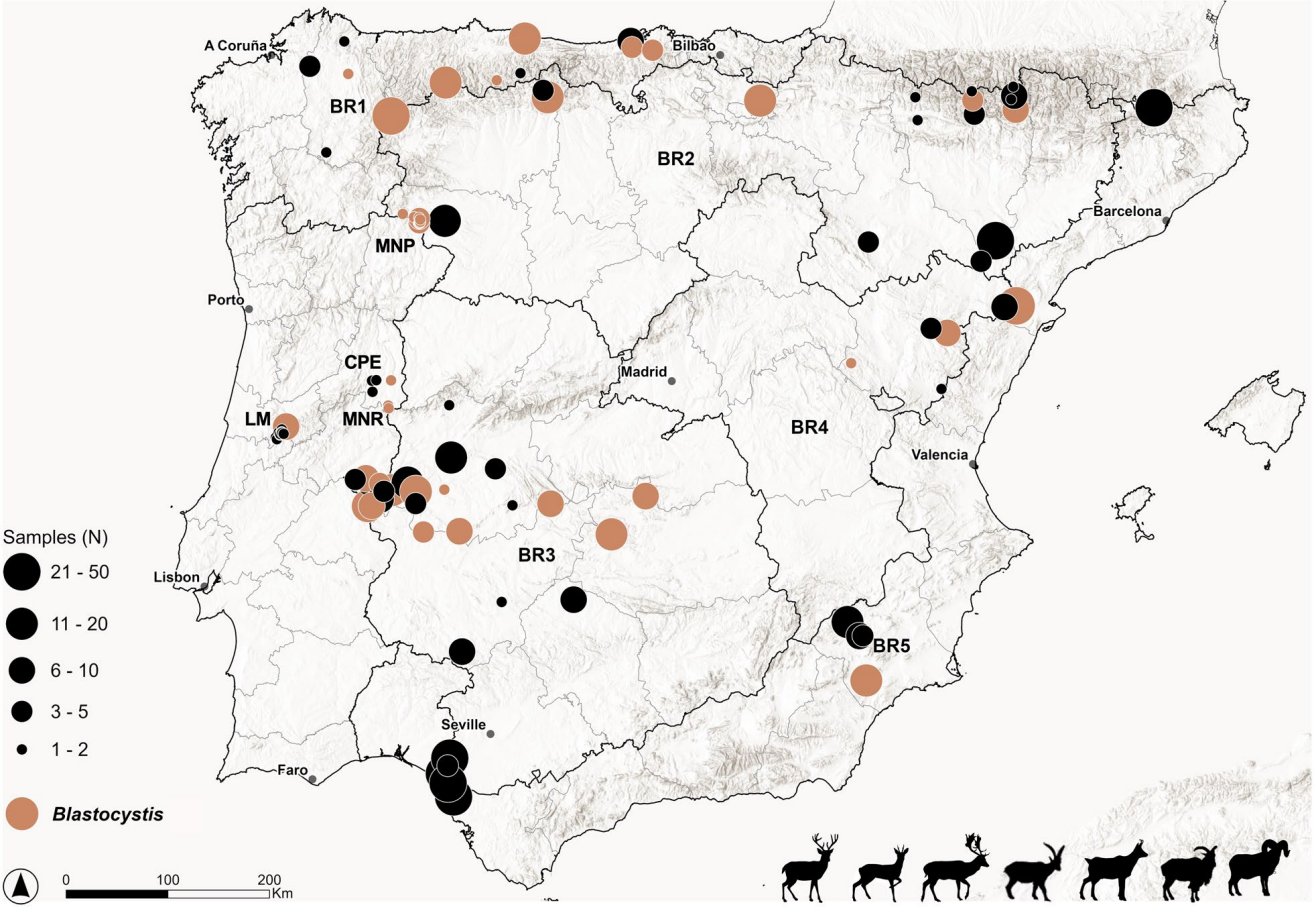
**Map of the Iberian Peninsula showing the sampling areas in Spain and Portugal.** The geographical distribution of *Blastocystis* detected in red deer (*Cervus elaphus*), roe deer (*Capreolus capreolus*), fallow deer (*Dama dama*), Iberian ibex (*Capra pyrenaica*), Southern chamois (*Rupicapra pyrenaica pyrenaica*), and Barbary sheep (*Ammotragus lervia*) is also indicated. MNP (Montesinho Natural Park), CPE (Central Portugal East), LM (Lousã Mountains), MNR (Malcata Nature Reserve).

A retrospective survey was performed using samples collected in the Iberian Peninsula between 1998 and 2021, encompassing 833 faecal samples from free-ranging wild ungulates in Spain (*n* = 699) and Portugal (*n* = 134). Frozen faecal samples from seven wild ruminant species in Spain (red deer, fallow deer*,* roe deer*,* Iberian ibex, Southern chamois*,* Barbary sheep, and mouflon) and two from Portugal (red deer and roe deer), collected across the five bioregions (BRs) of mainland Spain [39] and three comparable BRs in Portugal, were included in the study (Figure 1). Additional file 1, adapted from Muñoz et al. [40], summarises the features of the three adapted Portuguese BRs sampled in the present study and their corresponding locations (MNP–Montesinos Natural Park, LM–Lousã Mountains, West, CPE—Central Portugal East, and MNR–Malcata Nature Reserve).

The sampling was carried out in hunting estates, game reserves, natural areas, and other classified areas belonging to the European Union’s Natura 2000 Network sites [41]. Faecal samples were collected from legally hunted animals during field necropsies or from the ground while prospecting different transects throughout the sampling areas. Non-invasive samples were identified by experienced, field-trained personnel based on the morphology (e.g., shape, size, content) and deposition site. Each sample was placed in an individually labelled sterile tube, and the collection data and GPS location were recorded, when possible, before being stored at –20 °C by the responsible participating institution. Aliquots of each sample were then shipped to the Spanish National Centre for Microbiology (SNCM) in Majadahonda (Madrid, Spain) and the Department of Biology & CESAM (DBio & CESAM) at the University of Aveiro (Aveiro, Portugal) for sample processing.

### DNA extraction and purification

Genomic DNA was isolated from approximately 200 mg of each faecal sample using the QIAamp DNA Stool Mini Kit (Qiagen, Hilden, Germany), in accordance with the manufacturer’s instructions, except for the step where samples were mixed with the InhibitEX buffer, where the incubation time was changed to 10 min at 95 °C. Extracted and purified DNA samples were eluted in 200 µL of PCR-grade water and stored at 4 °C until further molecular analysis. The faecal DNA samples at the DBio & CESAM facilities were then shipped to the SNCM for subsequent molecular testing.

### *Blastocystis* screening

*Blastocystis* screening was initially performed using a direct PCR protocol targeting the small subunit ribosomal RNA (*ssu* rRNA) gene [42]. The amplification reaction encompassed a total volume of 25 µL, including 5 µL of template DNA and 0.5 µM of the primer pair BhRDr (5′–GAGCTTTTTAACTGCAACAACG–3′) and RD5 (5´–ATCTGGTTGATCCTGCCAGT–3′) to amplify a PCR product of ~600 bp. The reaction mix included 2.5 IU of MyTAQ™ DNA polymerase (Bioline GmbH, Luckenwalde, Germany) and a 5 × MyTAQ™ Reaction Buffer comprising 5 mM dNTPs and 15 mM MgCl_2_. PCR cycling conditions consisted of one step at 95 °C for 3 min, followed by 30 cycles of 1 min each at 94 °C, 59 °C, and 72 °C, with a final extension at 72 °C for 2 min, run on a 2720 thermocycler (Applied Biosystems, Foster City, CA). The resulting PCR amplicons were examined on 1.5% D5 agarose gels stained with Pronasafe (Conda, Madrid, Spain), together with positive and negative controls used in each PCR reaction and sized with a 100 bp DNA ladder (Boehringer Mannheim GmbH, Baden-Wurttemberg, Germany).

### *Blastocystis* subtype identification using next-generation amplicon sequencing

DNA aliquots of *Blastocystis*-positive samples, whose *ssu* rRNA-PCR amplicons produced bands on agarose gels compatible with the presence of *Blastocystis*, were shipped to the Environmental Microbial and Food Safety Laboratory, United States Department of Agriculture (Beltsville, Maryland, USA). Libraries for conducting next-generation amplicon sequencing (NGS) to identify *Blastocystis* subtypes were prepared as previously described [43]. Samples were subjected to PCR using primers ILMN_Blast505_532F and ILMN_Blast998_1017R, analogous to Blast505_532F/Blast998_1017R [44], except for containing Illumina overhang adapter sequences, amplifying a fragment of the *ssu* rRNA of ~500 bp. PCR products were detected using a QIAxcel (Qiagen, Valencia, CA, USA). A final pooled library concentration of 8 pM with 20% PhiX control was sequenced using Illumina MiSeq 600 cycle v3 chemistry (Illumina, San Diego, CA, USA). Paired ends were processed and analysed with an in-house pipeline that uses the BBTools package v39.00 [45], VSEARCH v2.27.1 [46], and BLAST + 2.14.0 +. After the removal of singletons, clustering and the assignment of centroid sequences to operational taxonomic units (OTU) was performed within each sample at a 98% identity threshold. Only OTUs containing a minimum of 100 sequences were retained. Raw FASTQ files were submitted to NCBI’s sequence read archive under the Bioproject PRJNA1219157. The nucleotide sequences obtained in this study have been deposited in GenBank under the accession numbers PV069227-PV069317.

### Generation of full-length *ssu* rRNA gene sequences

Three samples from roe deer (#P459, #P464, and #P465) observed to contain a potentially novel *Blastocystis* ST were used to obtain the full-length *ssu* rRNA gene nucleotide sequences required to validate novel subtypes. Full-length *ssu* rRNA gene sequences were generated using a previously described Nanopore-based sequencing strategy [19, 47]. Briefly, PCR was performed using MinION-tailed primers (forward: 5′-TTT CTG TTG GTG CTG ATATTG C AAC CTG GTT GAT CCT GCC AGT AGTC-3′; and reverse: 5′-ACT TGC CTG TCG CTC TATCTT C TGA TCC TTC TGC AGG TTC ACC TACG-3′) (MinION adapter nucleotide sequences under-lined) that amplify the full-length *ssu* rRNA gene sequence of most eukaryotic organisms. The sequencing library was prepared using Oxford Nanopore Technologies (Oxford, UK) SQK-LSK112/114 Ligation Sequencing Kit and EXP-PBC001 PCR Barcoding Kit following the manufacturer’s protocol for Ligation Sequencing Amplicons—PCR Barcoding (SQK-LSK114 with EXP-PBC001). The final library was loaded onto a MinION Flow Cell (R10.4.1) and sequenced on a MinION Mk1C. Following sequencing, reads were basecalled with dorado v0.8.2 using the model dna_r10.4.1_e8.2_400bps_sup@v5.0.0 and a minimum q-score cutoff of 10. Reads between a length of 1700 and 1900 nucleotides were retained, followed by dereplication and clustering at 98% identity using VSEARCH v2.27.1. Clusters of less than 10 reads were removed before they were filtered for chimeras using the vsearch –uchime_denovo command. Non-*Blastocystis* clusters were removed using the vsearch –usearch_global command (with flags –id 0.90 –query_cov 0.90) by aligning to a database of all ribosomal *Blastocystis* reference sequences available on NCBI (as of 09/24/24). Clusters that aligned to *Blastocystis* references were polished using Medaka v2.0.1 and their constituent reads and then re-clustered a final time. Final clusters were re-aligned to the same database as before using blastn v2.14.0 (with flags -task megablast -max_hsps 1 -evalue 1e-100 -perc_identity 85 -max_target_seqs 10 -word_size 64). The full-length nucleotide sequences of the *ssu* rRNA gene generated in this study were deposited in GenBank under the accession numbers PV065467-PV065470.

### Phylogenetic and pairwise distance analysis of full-length reference sequences

A phylogenetic tree was generated using the *Blastocystis* full-length *ssu* rRNA gene nucleotide sequences obtained in this study (samples #P459, #P464, and #P465) and reference nucleotide sequences for all currently validated STs obtained from GenBank. *Proteromonas lacertae*, a Stramenopile closely related to *Blastocystis*, was used as an outgroup. Sequences were aligned with the Clustal W algorithm, phylogenetic analyses performed using the neighbor-joining (NJ) method, and pairwise distances calculated with the Kimura 2-parameter model [48] using MEGA 11 [49]. This analysis involved 51 nucleotide sequences. All ambiguous positions were removed for each sequence pair (pairwise deletion option). There were a total of 1978 positions in the final dataset. Bootstrapping with 1000 replicates was used to determine support for the clades generated.

### Data analysis

*Blastocystis* prevalence was estimated in R software [50] using a binomial test to establish confidence limits with 95% confidence intervals (CIs). A *χ*^2^ test, using the chisq.test function was conducted to compare *Blastocystis* prevalence among different hosts, BRs, sampling sites, and sampling years. The bar plots were constructed using the ggplot2 package [51] in R software to illustrate the inter-subtype variation within Spanish and Portuguese wild ruminants, using the relative read abundances of each ST/ST subgroup. A different colour was assigned to each ST/ST subgroup identified.

## Results

### Occurrence of *Blastocystis*

A total of 833 faecal samples were collected across Spain (*n* = 699) and Portugal (*n* = 134) between 1998 and 2021 (Additional file 2). Overall, 13.8% (115/833; 95% CI 11.5–16.3) of the faecal samples from wild ruminants analysed were *Blastocystis*-positive by PCR and NGS. Samples that yielded PCR amplicons of the expected size but had no *Blastocystis* OTUs by NGS were conservatively considered negative. Wild ruminants from Portugal had higher *Blastocystis* carriage rates (38.1%, 51/134; 95% CI 29.8–468) than those from Spain (9.2%, 64/699; 95% CI 7.1–11.5) and this difference was statistically significant [*χ*^2^ (1, *n* = 833) = 49.6, *P* < 0.001].

Table 2 shows the distribution of *Blastocystis* in wild ruminants from Spain according to the sampling variables evaluated. A higher prevalence of *Blastocystis* was found in roe deer (12.9%, 12/93), red deer (11.9%, 39/329), and Barbary sheep (10.0%, 2/20), compared to Southern chamois (4.8%, 3/62), Iberian ibex (4.5%, 4/89), and fallow deer (4.2%, 4/96). None of the mouflon samples (*n* = 10) were *Blastocystis*-positive. Prevalence among the seven wild ruminant species analysed differed, although without reaching statistical significance [*χ*^2^ (6, *n* = 699) = 10.3, *P* = 0.114] (Table 2). *Blastocystis* occurrence also varied considerably among bioregions [χ^2^ (4, *n* = 699) = 47.3, *P* < 0.001], with animals from BR1 (23.2%) and BR3 (17.8%) presenting the highest prevalence. According to the sampling site, wild ruminants from hunting estates were more likely to harbour *Blastocystis* [*χ*^2^ (2, *n* = 699) = 19.1, *P* < 0.001]. In addition, *Blastocystis* occurrence was independent of the sampling period considered [*χ*^2^ (2, *n* = 699) = 1.2, *P* = 0.56].

**Table 2:** ***Blastocystis*** **carriage rates in Spanish free-ranging wild ruminant species according to the host, bioregion of origin, type of sampling site, and sampling year**

Variable	Samples (*n*)	*Blastocystis*-positive (*n*)	*Blastocystis*-positive (%)	95% CI (%)	*P*-value	Subtypes detected (*n*)
Host					0.114	
Red deer	329	39	11.9	8.6–15.8		**ST5** (1), **ST10a** (6), **ST10b** (9), **ST10**^**a**^ (3), ST13 (8), **ST14** (3), ST21 (3), **ST23** (3), ST24a (26), ST24b (20), ST24c (6), ST43 (1), ST44 (3)
Fallow deer	96	4	4.2	1.1–10.3		**ST5** (1), **ST10a** (3), **ST10b** (1), **ST14** (4), ST21 (2), ST24a (1), ST24b (1), ST24c (1), ST25 (1), ST26 (1), ST30 (1), ST42b (2), ST43 (1), ST44 (1)
Roe deer	93	12	12.9	6.8–21.5		**ST2** (1), **ST10a** (1), **ST10b** (2), ST13 (7), ST24a (1), ST24b (2), ST49 (2)
Iberian ibex	89	4	4.5	1.2–11.1		**ST2** (2), ST13 (3), ST24b (1)
Southern chamois	62	3	4.8	1.0–13.5		**ST5** (1), **ST10b** (2), **ST14** (1), ST21 (1), **ST23** (1), ST24a (1), ST24b (1), ST24c (1), ST44 (1)
Barbary sheep	20	2	10.0	1.2–31.7		**ST2** (1), ST24b (2)
Mouflon	10	0	–	–		–
Bioregion					** < 0.001**	
BR1	82	19	23.2	14.6–33.8		**ST5** (2), **ST10a** (5), **ST10b** (4), **ST10**^**a**^ (1), ST13 (7), **ST14** (4), ST21 (5), **ST23** (4), ST24a (4), ST24b (7), ST24c (5), ST25 (1), ST26 (1), ST30 (1), ST42b (2), ST43 (1), ST44 (3), ST49 (2)
BR2	125	5	4.0	1.3–9.1		**ST2** (1), **ST10b** (3), **ST10**^**a**^ (1), **ST14** (1), ST24a (3), ST24b (1)
BR3	185	33	17.8	12.6–24.1		**ST5** (1), **ST10a** (5), **ST10b** (6), **ST10**^**a**^ (1), ST13 (8), **ST14** (3), ST21 (1), ST24a (24), ST24b (16), ST24c (3), ST43 (1), ST44 (2)
BR4	24	2	8.3	1.0–27.0		**ST2** (1), **ST10b** (1), ST13 (1)
BR5	283	5	1.8	0.6–4.1		**ST2** (2), ST13 (2), ST24b (3)
Type of sampling site					** < 0.001**	
Hunting estates	341	42	12.3	9.0–16.3		**ST2** (3), **ST5** (1), **ST10a** (5), **ST10b** (7), **ST10**^**a**^ (1), ST13 (10), ST14 (4), ST21 (1), ST24a (27), ST24b (18), ST24c (3), ST43 (1), ST44 (1), ST49 (2)
Game reserve	195	22	11.3	7.2–16.6		**ST2** (1), **ST5** (2), **ST10a** (5), **ST10b** (7), **ST10**^**a**^ (2), ST13 (8), **ST14** (4), ST21 (5), **ST23** (4), ST24a (3), ST24b (9), ST24c (5), ST25 (1), ST26 (1), ST30 (1), ST42b (2), ST43 (1), ST44 (3)
Natural protected area	163	0	–	–		
Sampling year^**b**^					0.56	
1998–2001	58	7	12.1	5.0–23.3		**ST10a** (1), ST13 (6), ST24b (2)
2011–2012/2014–2015	13	2	15.4	1.9–45.4		**ST2** (1), **ST10b** (1), **ST14** (1), ST24a (1)
2018–2021	606	52	8.6	6.5–11.1		**ST2** (3), **ST5** (2), **ST10a** (9), **ST10b** (11), **ST10**^**a**^ (2), ST13 (12), ST14 (7), ST21 (5), **ST23** (3), ST24a (27), ST24b (25), ST24c (7), ST25 (1), ST26 (1), ST30 (1), ST42b (2), ST43 (2), ST44 (4), ST49 (2)

Only samples confirmed to contain *Blastocystis* by next-generation amplicon sequencing (NGS) were considered positive

95% Confidence Intervals (95% CI) are included. Values in bold represent statistical significance

^a^Potential novel ST10 subgroup based on sequence similarity and phylogenetic clustering

^b^Twenty-two samples with unknown sampling year were excluded from the analysis. Two of them belonged to red deer and carried subtypes **ST10b**, **ST10**^**a**^ and ST24a. One of them belonged to Southern chamois and carried subtypes **ST5**, **ST10b**, ST21, ST23, ST24a, ST24c, and ST44.

Table 3 shows the distribution of *Blastocystis* in wild ruminants from Portugal. All investigated animals were from natural classified areas. Red deer displayed a higher *Blastocystis* prevalence (40.4%, 38/94) than that found in roe deer (32.5%, 13/40), although this difference was not statistically significant [*χ*^2^ (1, *n* = 134) = 0.2, *P* = 0.686]. Neither bioregion [*χ*^2^ (2, *n* = 134) = 0.2, *P* = 0.895] nor sampling year [*χ*^2^ (3, *n* = 134) = 0.3, *P* = 0.960] influenced the occurrence of *Blastocystis* in the two wild ruminant species investigated.

**Table 3:** ***Blastocystis*** **carriage rates in Portuguese free-ranging wild ruminant species according to the bioregion of origin, type of sampling site, and sampling year**

Variable	Samples (*n*)	*Blastocystis* positive (*n*)	*Blastocystis*- positive (%)	95% CI (%)	*P*-value	Subtypes detected (*n*)
Host					0.686	
Red deer	94	38	40.4	30.4–51.0		**ST2** (1), **ST5** (4), **ST10b** (5), ST21 (5), ST24a (12), ST24b (32), ST24c (13), ST25 (1)
Roe deer	40	13	32.5	18.6–49.1		**ST5** (2), **ST10b** (1), ST13 (10), ST44 (1), ST49 (3)
Bioregion					0.895	
BR1	52	18	34.6	22.0–49.1		**ST2** (1), **ST5** (3), ST24b (13), ST24c (13)
BR2	56	22	57.1	43.2–70.3		**ST5** (1), **ST10b** (5), ST13 (2), ST21 (5), ST24a (12), ST24b (19), ST25 (1)
BR3	26	11	42.3	23.4–63.1		**ST5** (2), **ST10b** (1), ST13 (8), ST44 (1), ST49 (3)
Type of sampling site					NA	
Natural protected area	134	51	38.1	29.8–46.8		**ST2** (1), **ST5** (6), **ST10b** (6), ST13 (10), ST21 (5), ST24a (12), ST24b (32), ST24c (13), ST25 (1), ST44 (1), ST49 (3)
Sampling year					0.960	
2017	18	8	44.4	21.5–69.2		**ST10b** (3), ST21 (3), ST24a (5), ST24b (8)
2019	15	5	33.3	11.8–61.6		**ST2** (1), **ST5** (1), **ST10b** (1), ST24b (4), ST44 (1)
2020	80	31	38.8	28.1–50.3		**ST5** (2), **ST10b** (2), ST13 (6), ST21 (2), ST24a (7), ST24b (19), ST24c (13), ST25 (1), ST49 (1)
2021	21	7	33.3	14.6–60.0		ST5 (3), ST13 (4), ST24b (1), ST49 (2)

NA, not applicable

Only samples confirmed to contain *Blastocystis* by next-generation amplicon sequencing (NGS) were considered positive

95% Confidence Intervals (95% CI) are included

BR1 encompasses Lousã Mountains (LM), BR2 Montesinho Natural Park (MNP) and BR3 Central Portugal East (CPE) and Malcata Nature Reserve (MNR). Values in bold represent statistical significance.

### Molecular characterisation of *Blastocystis* using NGS

Fourteen known *Blastocystis* subtypes (ST2, ST5, ST10, ST13, ST14, ST21, ST23, ST24-ST26, ST30, ST42-ST44) and a novel subtype (named ST49) were identified among the 64 *Blastocystis*-positive samples from Spain (Table 2 and Additional file 2). Two previously recognised subgroups of ST10 (ST10a and ST10b) plus a potentially novel subgroup for this ST (based on sequence similarity and phylogenetic clustering) were identified. The three subgroups currently recognised for ST24 (ST24a-ST24c) were identified. Eleven STs were identified in fallow deer, nine in red deer, seven in Southern chamois, five in roe deer, three in Iberian ibexes and two in Barbary sheep (Table 2 and Additional file 3). According to our results, ST24a was the most prevalent *Blastocystis* ST identified (46.9%, 30/64), detected in red deer, roe deer, fallow deer, and Southern chamois, followed by ST24b (42.2%, 27/64), observed in all six wild ruminants positive for *Blastocystis*, and ST13 (28.1%, 18/64), recorded in red deer, roe deer, and Iberian ibex (Table 2 and Additional file 3). However, the most prevalent ST differed among hosts. In red deer, ST24a was the most common *Blastocystis* ST detected (66.7%, 26/39), followed by ST24b (51.3%, 20/39), ST10b (23.1%, 9/39) and ST13 (20.5%, 8/39). In fallow deer, ST14 (100%, 4/4) and ST10a (75%, 3/4) were the most prevalent STs detected. ST13 was the predominant ST circulating in roe deer (58.3%, 7/12) and Iberian ibex (75%, 3/4). In Southern chamois, ST10b (66.7% 2/3) was the most common ST, while in Barbary sheep, it was ST24b (100%, 2/2) (Table 2). One zoonotic ST (ST2) and four potentially zoonotic STs (ST5, ST10, ST14, and ST23) were identified in the six Spanish wild ruminant hosts investigated. Potentially zoonotic STs were present in all *Blastocystis*-positive samples from fallow deer and Southern chamois, 50.0% from Iberian ibexes and Barbary sheep, 43.6% from red deer, and 25.0% from roe deer (Table 2 and Additional file 2).

Eight STs (ST2, ST5, ST10b, ST13, ST21, ST24a/b/c, ST25, and ST44) and the novel ST49 were identified among the 51 NGS *Blastocystis*-positive samples from Portugal (Table 3, and Additional file 2). Six STs were identified in red deer, while in roe deer, there were five (Table 3 and Additional file 3). ST24 (subgroup ST24b: 84.2%, 32/38; ST24c: 34.2%, 13/38; and ST24a: 31.6%, 12/38) was only detected in red deer in Portugal being the most prevalent ST identified in this host. On the other hand, ST13 was the most prevalent ST identified in roe deer (76.9%, 10/13) but not detected in red deer (Table 3 and Additional file 3). Zoonotic (ST2) and potentially zoonotic (ST5 and ST10b) STs were present in 26.3% (10/38) and 23.1% (3/13) of the *Blastocystis*-positive samples from red and roe deer, respectively.

### *Blastocystis* mixed ST infections and intra subtype diversity by NGS

**Figure 2:**
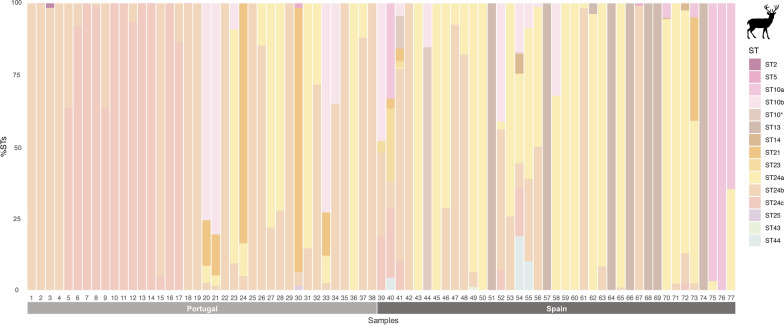
**Relative abundance of each *Blastocystis* ST detected in Spanish and Portuguese red deer (*Cervus elaphus*).** A colour was attributed to each ST or ST subgroup identified.

**Figure 3:**
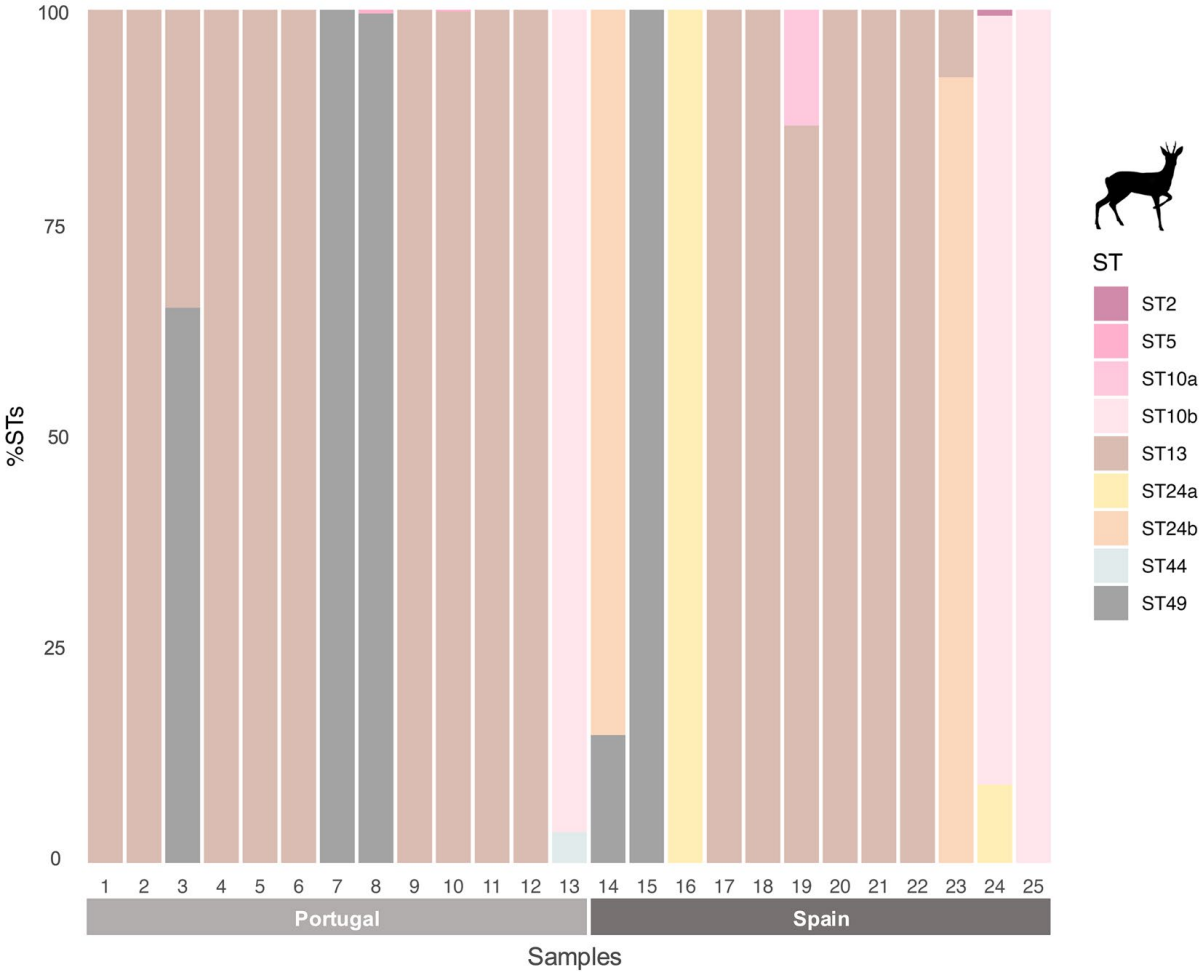
**Relative abundance of each *Blastocystis* ST detected in Spanish and Portuguese roe deer (*Capreolus capreolus*)**. A colour was attributed to each ST or ST subgroup identified.

**Figure 4:**
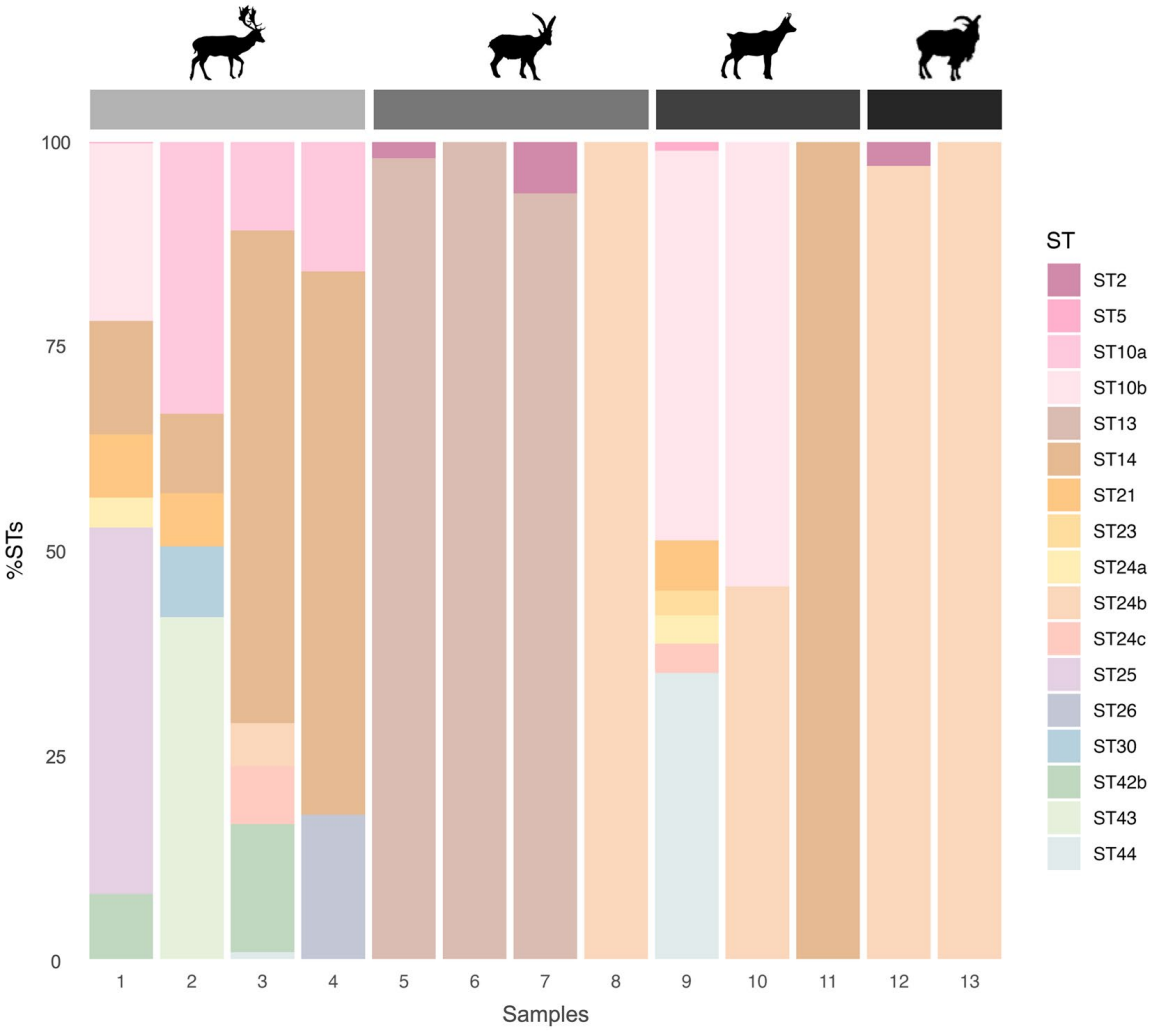
**Relative abundance of each *Blastocystis* ST detected in Spanish and Portuguese wild ruminants.** From left to right species icons correspond to fallow deer (*Dama dama*), Iberian ibex (*Capra pyrenaica*), Southern chamois (*Rupicapra pyrenaica pyrenaica*), and Barbary sheep (*Ammotragus lervia*). A colour was attributed to each ST or ST subgroup identified.

In Spain, mixed ST infections containing 2–7 distinct STs were found in over half of the total *Blastocystis*-positive samples (60.9%, 39/64). Seven different STs were observed in four samples from two red deer, one fallow deer, and one Southern chamois (Figures 2, 4, and Additional file 2). Roe deer had the highest frequency of mono-infections (66.7%, 8/12), while 100% of the *Blastocystis*-positive samples from fallow deer presented mixed infections of three or more STs (Figures 3 and 4).

In Portugal, a similar pattern to the one reported in Spain was observed, with mixed ST infections found in over half of the total *Blastocystis-*positive samples (54.9%, 28/51), being more common in red deer (63.2%, 24/38) than in roe deer (30.8%, 4/13). Most of the mixed infections encompassed a combination of two STs (41.2%, 21/51), while co-infections by three or four distinct STs were identified only in red deer samples (Figures 2, 3, and Additional file 2).

Intra-subtype diversity was observed in seven STs (ST2, ST5, ST10a/b, ST14, ST21, ST24a/b/c, and ST44). ST14 and ST subgroups, ST24b, ST24a, ST10b, exhibited the highest intra-subtype diversity (seven unique genetic variants for ST24b, six for ST24a and five for ST10b and ST14), followed by ST5 and ST21 (four genetic variants each), ST2, ST24c and ST44 (three genetic variants each), and ST10a (two unique genetic variants) (Additional files 2 and 4).

### Host preference

A direct comparison of the occurrence and relative abundance of *Blastocystis* ST/subgroup found within each of the investigated Spanish and Portuguese wild ruminant species revealed distinct ST patterns in host preference. In Spanish roe deer, ST13 presented the highest prevalence (58.3%, 7/12) (Table 4). Reads for ST13 represented a high percentage among the reads of the roe deer positive for ST13 (84.9%; range of 7.9–100%), including mono-infections in five roe deer (Figure 3 and Table 4). The novel ST49 was exclusively found in roe deer (16.7%, 2/12), either in co-infection with ST24b (with a relative abundance of 15.0%) or as a mono-infection. ST13 was also the most frequent ST in the Iberian ibex, detected in 75.0% of the *Blastocystis*-positive samples at high relative abundances (93.7–100%) (Figure 4 and Table 4). For red deer, the most frequently identified ST was subgroup ST24a (66.7%, 26/39; relative abundance: 0.5–100%), including four mono-infections. Other common STs/ST subgroups found in Spanish red deer were ST24b (51.3%, 20/39; relative abundance: 0.9–100%), ST10b (23.1%, 9/39; relative abundance: 0.3–48.0%), and ST13 (20.5%, 8/39; relative abundance: 3.7–100%). ST24b was also the most frequent *Blastocystis* ST in Barbary sheep, detected in 100% of the *Blastocystis*-positive samples, either in co-infection with ST2 (relative abundance: 97.1% vs 2.9%) or as a mono-infection. For fallow deer, ST14 was the predominant ST detected, present in 100% of the *Blastocystis*-positive samples, with relative abundances ranging from 9.7% to 66.5%. ST25, ST26, ST30, and subgroup ST42b were exclusively found in fallow deer, the latter detected in two samples at moderate-to-low relative abundance (8.0–15.7%). ST10b was also the predominant ST in Southern chamois (66.7%, 2/3; relative abundance: 54.4%) (Figure 4, Table 4, and Additional file 2).

**Table 4:** **Occurrence of** ***Blastocystis*** **subtypes and mean and range of sequencing reads attributed to each subtype detected for free-ranging wild ruminant species in Spain using next-generation amplicon sequencing (NGS)**

Subtype	Subtype prevalence (%)						Subtype reads (mean, %)						Subtype range (range, %)					
	Red deer	Fallow deer	Roe deer	Iberian ibex	Southern chamois	Barbary sheep	Red deer	Fallow deer	Roe deer	Iberian ibex	Southern chamois	Barbary sheep	Red deer	Fallow deer	Roe deer	Iberian ibex	Southern chamois	Barbary sheep
**ST2**	0	0	8.3	50	0	50	–	–	0.7	4.1	–	3.0	–	–	0.7	2.0–6.3	–	3.0
**ST5**	2.6	25	0	0	33.3	0	0.9	0.2	–	–	1.2	–	0.9	0.2	–	–	1.2	–
**ST10a**	15.4	75	8.3	0	0	0	50.8	20.0	13.6	–	–	–	4.9–100^**b**^	10.8–33.3	13.6	–	–	–
**ST10b**	23.1	25	16.7	0	66.7	0	18.7	21.7	95.1	–	54.4	–	0.3–48.0	21.7	90.1–100^**b**^	–	54.4	–
**ST10**^**a**^	7.7	0	0	0	0	0	32.3	–	–	–	–	–	0.6–84.7	–	–	–	–	–
ST13	20.5	0	58.3	100	0	0	88.0	–	84.9	97.2	–	–	3.7–100^**b**^	–	7.9–100^**b**^	93.7–100^**b**^	–	–
**ST14**	7.7	100	0	0	33.3	0	3.2	37.6	–	–	100	–	0.4–6.8	9.7–66.5	–	–	100^**b**^	–
ST21	7.7	50	0	0	33.3	0	14.6	7.1	–	–	7.0	–	3.5–36.1	6.4–7.7	–	–	7.0	–
**ST23**	7.7	0	0	0	33.3	0	7.8	–	–	–	3.4	–	2.1–25.2	–	–	–	3.4	–
ST24a	66.7	25	8.3	0	33.3	0	62.7	3.7	54.6	–	4.0	–	0.5–100^**b**^	3.7	9.2–100^**b**^	–	4.0	–
ST24b	51.3	25	16.7	24	33.3	100	39.9	5.3	88.6	100	45.7	98.5	0.9–100^**b**^	5.3	85.0–92.1	100^**b**^	45.7	97.1–100^**b**^
ST24c	15.4	25	0	0	33.3	0	13.1	7.1	–	–	4.1	–	2.3–24.3	7.1	–	–	4.1	–
ST25	0	25	0	0	0	0	–	44.8	–	–	–	–	–	44.8	–	–	–	–
ST26	0	25	0	0	0	0	–	17.7	–	–	–	–	–	17.7	–	–	–	–
ST30	0	25	0	0	0	0	–	8.7	–	–	–	–	–	8.7	–	–	–	–
ST42b	0	50	0	0	0	0	–	11.8	–	–	–	–	–	8.0–15.7	–	–	–	–
ST43	2.7	25	0	0	0	0	1.1	41.9	–	–	–	–	1.1	41.9	–	–	–	–
ST44	7.7	25	0	0	0	0	11.0	0.9	–	–	40.0	–	4.2–18.7	0.9	–	–	40.0	–
ST49	0	0	16.7	0	0	0	–	–	57.5	–	–	–	–	–	15.0–100^**b**^	–	–	–

Potentially zoonotic subtypes are in bold

^a^Potential novel ST10 subgroup based on sequence similarity and phylogenetic clustering

^b^A 100 value represents mono-infection.

In the Portuguese red deer populations, subgroup ST24b was the most frequently identified ST (84.2%, 32/38) at variable relative abundances ranging from 0.4% to 100%, eight as a mono-infection (Figure 2 and Table 5). ST24c was the second most prevalent ST detected in this host (34.2%, 13/38), also at variable relative abundances (4.6–100%), including five mono-infections. ST5 was detected in 10.5% (4/38) of the red deer samples at very low relative abundances (0.1–1.6%). Like in the case of the Spanish populations, ST13 was the predominant ST detected in Portuguese roe deer (76.9%, 10/13; relative abundance: 35.7–100%), including eight mono-infections. Novel subtype ST49 was the second most frequently detected (and exclusively found) *Blastocystis* ST in this host species (23.1%, 3/13; relative abundance: 65.1–100%), including one mono-infection (Figure 3, Table 5, and Additional file 2).

**Table 5:** **Occurrence of** ***Blastocystis*** **subtypes and mean and range of sequencing reads attributed to each subtype detected for free-ranging wild ruminant species in Portugal using next-generation amplicon sequencing (NGS) in the present study**

Subtype	Subtype prevalence (%)		Subtype reads (mean, %)		Subtype reads (range, %)	
	Red deer	Roe deer	Red deer	Roe deer	Red deer	Roe deer
**ST2**	2.6	0	1.5	–	1.5	–
**ST5**	10.5	15.4	2.1	0.3	0.2–1.6	0.2–0.4
**ST10b**	13.2	7.7	54.7	96.4	9.1–80.6	96.4
ST13	0	76.9	–	93.5	–	35.7–100^a^
ST21	13.2	0	44.2	–	15.9–92.2	–
ST24a	31.6	0	41.9	–	3.8–100^a^	–
ST24b	84.2	0	53.0	–	0.4–100^a^	–
ST24c	34.2	0	84.7	–	4.6–100^a^	–
ST25	2.6	0	1.4	–	1.4	–
ST44	0	7.7	–	3.6	–	3.6
ST49	0	23.1	–	88.2	–	65.1–100^a^

Potentially zoonotic subtypes/subtype groups are in bold

^a^A 100 value represents mono-infection.

### Validation of novel subtype ST49

Nucleotide sequences for the novel ST49 generated for the Santin region by Illumina were compared to nucleotide sequences available in the GenBank database. The closest match to *Blastocystis* sequences available in GenBank was 100% to unpublished *Blastocystis* nucleotide sequences with no subtype information that were obtained from 12 Korean water deer (*Hydropotes inermis argyropus*) faecal samples from South Korea (MT114836-MT114847). The closest nucleotide sequences for a currently recognised subtype available in GenBank was ST31 from samples obtained from two white-tailed deer (*Odocoileus virginianus*) faecal samples from the USA (MZ267637 and MZ267676) with 97.2% and 97.7% similarity, respectively. A Nanopore sequencing strategy was employed to obtain the full-length nucleotide sequence of the *ssu* rRNA gene from three roe deer samples (#P459, #P464, and #P465) containing the novel subtype. ST49 full-length nucleotide sequences of the *ssu* rRNA gene were obtained for all three samples. Additionally, for sample #P459, a ST13 full-length nucleotide sequence of the *ssu* rRNA gene was also obtained (detected also by NGS using Illumina sequencing). This ST13 nucleotide sequence has a 99.9% similarity with a ST13 sequence obtained from a mouse deer (*Tragulus javanicus*) from the UK (KC148209).

**Figure 5:**
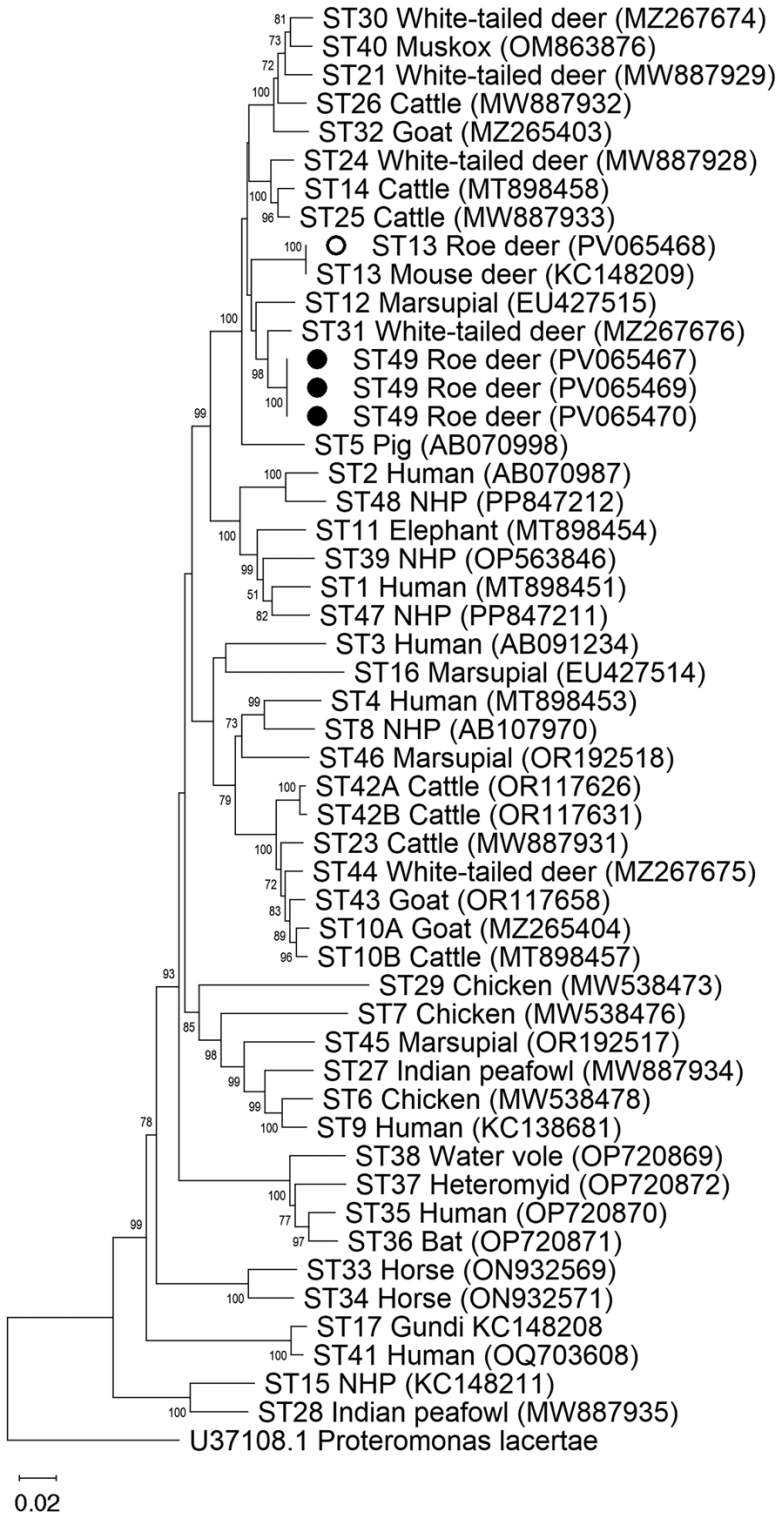
**Phylogenetic relationships among *Blastocystis* full-length *ssu* rRNA gene nucleotide sequences.** Sequences generated in the present study are represented with circles (black filled for ST49 sequences). *Proteromonas lacertae* was used as outgroup taxon to artificially root the tree. Analysis was inferred using the neighbour-joining method with genetic distances calculated using the Kimura 2-parameter model. This analysis involved 51 nucleotide sequences, and there were a total of 1978 positions in the final dataset. Bootstrap values lower than 75% were not displayed.

Phylogenetic analysis of full-length sequences using the NJ method demonstrated that novel ST49 clustered with ST31 with bootstrap support of 98 in a cluster that also included ST12 and ST13 (Figure 5). Pairwise distance comparisons were used to evaluate the percentage of shared sequence identity of ST49 with known subtypes. The highest percentage of sequence similarity for ST49 was with ST31 (98%). Together, these data support the designation of this novel sequence to ST49.

## Discussion

An expansion of *Blastocystis* subtype diversity is pronounced with the increased number of surveys taking place in new hosts and geographic regions of the world. The number of valid subtypes has increased from 25 in 2021 (ST1–ST17, ST21, and ST23–ST29) [52] to the 44 currently validated as of 2024 (ST1–ST17, ST21, ST23–ST48) [19, 22, 23]. However, there are still important knowledge gaps regarding host specificity and range for *Blastocystis* genetic variants. To get a clear picture of the current epidemiology, the screening of different host species from diverse world locations using reliable tools and harmonised procedures is needed [29]. The ambitious, molecular-based epidemiological survey conducted in this study represents the first attempt to assess the occurrence, subtype diversity, and zoonotic potential of *Blastocystis* in wild ruminants in the Iberian Peninsula.

*Blastocystis* was found in Iberian wild ruminants with an overall prevalence of 13.8%; however, a significantly higher prevalence was detected in Portuguese populations compared with their Spanish counterparts (38.1% vs. 9.2%, respectively). This difference withstands even when only red deer and roe deer, the two wild ruminants examined in both locations, are considered (Tables 2 and 3). The prevalence of *Blastocystis* in Spanish red deer and roe deer was 11.9% (39/329) and 12.9% (12/93), respectively, while the prevalence for red deer and roe deer from Portugal was 40.4% (38/94) and 32.5% (13/40), respectively. The 11.9% prevalence found across the sampled populations of Spanish red deer was within the range (2–27%) previously reported for wild populations in this host species from Australia and Poland [22, 30, 34]. In contrast, the prevalence found in Portuguese red deer (40.4%) was the highest reported globally in free-ranging wild red deer (Table 1). *Blastocystis* has also been identified in two surveys conducted in captive red deer from China (33.3–40%) [31, 32] and the United Kingdom (33.3–100%) [33]. The same pattern was observed for roe deer, where *Blastocystis* was more prevalent in Portuguese populations than in the Spanish ones (32.5% vs. 12.9%, respectively). No previous surveys have reported the presence of *Blastocystis* in wild roe deer, although infections ranging from sporadic to frequent (50.0%) have been documented in a limited number of zoo captive animals from Denmark [35] and the United Kingdom [36].

Concerning the remaining wild ruminants sampled in Spain, this study represents the first report of *Blastocystis* in Iberian ibexes (4.5%) and Southern chamois (4.8%) as there are no records of the presence of the protist in wild or captive animals of these two host species. *Blastocystis* in free-ranging fallow deer was previously described in wild populations from Australia with a higher prevalence (63.3%) than the one found in this study (4.2%). *Blastocystis* has also been previously found in captive fallow deer from China (31–50%) [30, 37], Mauritius (100%) [36], and Italy [38], although most of these studies investigated a very limited number of animals (*n* = 1–2). The finding of *Blastocystis* in free-ranging Barbary sheep (10%) is also novel, as it had only been described in captive animals so far. Specifically, in the only two studies conducted to date in captive Barbary sheep from China and Libya, the protist was identified in 100% and 20% of the samples, respectively [36, 37]. Finally, none of the ten mouflon samples analysed were positive for *Blastocystis*, although it has been previously reported in a single captive specimen from the Czech Republic [36].

Our NGS analyses confirmed the presence of fifteen *Blastocystis* STs (ST2, ST5, ST10, ST13, ST14, ST21–26, ST30, ST42–44), including a novel one (ST49) circulating within the Iberian wild ruminant populations under investigation. Full-length *ssu* rRNA reference nucleotide sequences obtained in this study support the designation of the novel subtype named ST49. Five *Blastocystis* nucleotide sequences from roe deer, including three from Portugal and one from Spain, were identified as likely novel based on their low sequence similarity with any known ST at the Santin region obtained by NGS. Full-length *ssu* rRNA reference nucleotide sequences were obtained employing an Oxford Nanopore sequencing strategy from three of those samples and analysed to validate the identification of these novel sequences in accordance with current recommendations for *Blastocystis* nomenclature [19, 53]. These sequences were compared to other full-length reference sequences from accepted subtypes of *Blastocystis* (ST1–ST17, ST21, ST23–ST48) to determine if phylogenetic analysis and pairwise sequence comparison support their designation as new subtypes. Phylogenetic analysis demonstrated strong support for the branching of ST49 with ST31 with bootstrap support of 98 (Figure 5). Pairwise comparison of full-length sequences also demonstrated that ST49 shares ≤ 97% sequence similarity with any known subtype. These data support the designation of ST49 as a new subtype that nests within a clade composed mainly of subtypes commonly observed in ruminants. Although strict host specificity appears to be rare within the species, these findings are beneficial in advancing our understanding of host preference among genetic variants of *Blastocystis*.

A higher genetic diversity (15 STs *vs*. 9 STs) and mixed infection rates (60.9% vs. 54.9%) were found in the wild ruminants from Spain than those from Portugal. This is interesting, considering that prevalence was significantly higher in wild ruminants from Portugal (38.1%) than those from Spain (9.2%). When only the two host species, red deer and roe deer, examined in both countries are considered, those differences are still clear for the red deer, for which six STs were detected in Portugal versus the nine detected in the Spanish red deer. However, five STs were detected in roe deer from Spain and Portugal. Although we cannot be certain about the reasons for the ST differences between Portuguese and Spanish red deer, we believe the differences in sampling sites (hunting estates and game reserves in Spain versus natural protected areas in Portugal) may have influenced these results (Additional file 5). This is likely due to common practices in Mediterranean hunting estates/game reserves, such as supplemental feeding to maintain artificially high population densities (especially in BR3), which increases the risk of disease transmission in wild ruminants due to aggregation behaviours (Additional file 5) [54].

Considering that only red deer and roe deer were sampled in Portugal, future studies should explore genetic variability in other wild ruminant host species inhabiting Portugal, including fallow deer and the Iberian ibex. Of the fifteen *Blastocystis* STs detected, six STs (ST10, ST14, ST21, and ST23-ST25) were previously reported in wild populations of red deer and fallow deer from Australia (Table 1). In contrast, only ST5 and ST10 have been previously reported in the remaining studies from the same free-ranging/captive ruminant species worldwide, while ST1 and ST4 (previously identified in free-living red deer) were not detected in the present survey (Tables 1, 2, and 3). Zoonotic (ST2) or potentially zoonotic (ST5, ST10, ST14, and ST23) STs were found in 37.4% (43/115) of all *Blastocystis*-positive samples. The increasing wild ungulate expansion, predominantly in overlapped habitats with free-ranging livestock or suburban areas, may pose a risk of transmitting these potentially zoonotic STs to humans. This is particularly important when considering ungulate species-specific traits, including feeding or social behaviours. For instance, the fallow deer, which harboured at least two potentially zoonotic STs, is regarded as a grazer, more dependent on pastures, and a highly gregarious species [55]. These traits might increase the infection burden [55], raising the chances of cross-transmission events with other wild or domestic animals and humans. Nevertheless, it is important to state that some of the potentially zoonotic *Blastocystis* subtypes identified in the present study, concretely ST2 (frequent in humans) and ST5 (identified sporadically in humans but more frequently in wild and domestic swine), were found at low relative abundances (0.2–6.3%). This raises the question of whether the presence of these STs results from mechanical carriage after accidental ingestion of the protist via contaminated food or water, as previously described in free-ranging and captive wild carnivores [56].

According to Hublin et al. [14], ST10 and ST14 are the two most frequently identified STs in wild ungulates, including cervid, ovine, and caprine species. However, these two subtypes, together with ST42b, ST43, and ST44 (previously known as ST10e, ST10f, and ST10c, respectively; see reference [19]), were only found in 28.7% (33/115) of all *Blastocystis*-positive samples (Tables 2 and 3). ST10b and ST14 were indeed the most common STs identified in Southern chamois (66.7%, 2/3) and fallow deer (100%, 4/4), respectively; nevertheless, ST24a/b/c was the ST most frequently detected in Barbary sheep (100%, 2/2) and red deer (76.9%, 30/39) populations from Spain and Portugal (100%, 38/38). Interestingly, even though ST25 and ST26 have been reported as the predominant STs in cattle from Spain [57] and Portugal [58], these two STs were rarely observed in the wild ruminant samples in the present study (Tables 2 and 3). In comparison, ST10a/b, ST14, and ST24a/b/c were also frequently identified within cattle and small ruminant livestock species and in the wild ruminants investigated in the present study. This finding might be indicative of potential cross-species transmission between wild and domestic hosts sharing habitats, either due to direct contact or through environmental contamination of pastures or surface waters. Supporting this hypothesis, the same unique genetic variant associated with ST24b detected in red deer samples from MNP in this survey has previously been found circulating in sheep from northeast Portugal [58]. Another interesting observation regarding ST24 subgroup variation was that the red deer population from MNP was exclusively infected with ST24a/ST24b (100%, 20/20), while the red deer population from LM was infected with ST24b/ST24c (100%, 18/18). While ST24b was found circulating in both populations, ST24a was only detected in MNP and ST24c in the LM sampling area (Additional file 5). These findings appear to suggest some degree of ST24 subgroup specificity within the two sampled red deer populations. This hypothesis is strengthened by the fact that ST24a/b/c are the most prevalent ST detected in our Portuguese red deer population (Figure 2, Tables 3, and 5). An explanation for the ST24 variation may be related to differences in the environmental conditions of each sampling site (LM is located within BR1 and MNP within BR2) and the presence/absence of free-roaming livestock herds (free-roaming livestock herds are not a common practice in LM, while they are frequent in MNP) (Additional file 5).

*Blastocystis* ST diversity in roe deer was particularly interesting, as ST13 was the predominant ST identified in both the Spanish (58.3%; relative abundance: 7.9–100%) and Portuguese populations (76.9%; relative abundance: 35.7–100%) (Figure 3, Tables 2, 3, 4, and 5). This constitutes the first report of this ST in roe deer as ST13 has only been previously reported in cattle from Portugal [58], mouse deer from UK [36], quokka (*Setonix brachyurus*) from Australia [59], and an unpublished report in goats from China (OQ727440). Only one unique genetic variant was found circulating within the investigated roe deer populations, regardless of the country of origin (Additional file 4). Close geographical proximity between the Portuguese (MNP and MNR) and Spanish roe deer populations might increase the likelihood of ST13 cross-transmission among infected individuals and explain, at least partially, this finding. Remarkably, novel subtype ST49 was only detected in roe deer specimens from one region of Portugal (MNR) and Spain (Cantabria–BR1) (Figs. 1, 3, and Additional files 2, 6). This ST shows a close phylogenetic relationship with ST31, first identified in white-tailed deer from the USA [60], suggesting potential host preference of ST31 and ST49 towards wild cervid species. This is also supported by the identification of nucleotide sequences identical to ST49 in 12 Korean water deer (unpublished; GenBank Accession numbers MT114836-MT114847).

Zoonotic ST2, along with ST13 and ST24b, were the only three *Blastocystis* found in the Iberian ibex. Likewise, the very same ST13 unique genetic variant found circulating in roe deer populations from Spain and Portugal was found in this host and two different sampling areas (Teruel–BR4 and Tarragona–BR5) (Figures 1, 4, Additional files 2, and 7). This finding may be indicative of some degree of host specificity but also of a broader geographic spread and high environmental plasticity, considering the diversity of locations and bioregions where it was detected.

This study has some design limitations that might have biased some of the results obtained and the conclusions reached. First, most faecal samples available from Spanish wild ungulate species were stored for long periods prior to analysis. Despite freezing conditions, the prolonged time elapsed between sample collection and DNA extraction might have compromised the performance of the molecular methods used for detection and genotyping purposes, contributing to the lower prevalence rates observed in Spain. Second, opportunistic sampling during legal hunting periods may have biased geographical and temporal representativeness.

In conclusion, this is the first NGS-based study conducted in the Iberian Peninsula to characterise *Blastocystis* subtype diversity in wild ruminants and the largest epidemiological survey investigating the occurrence of this protist in such wild hosts carried out globally. The use of NGS has enabled us to capture not only the broad molecular diversity of *Blastocystis* (including mixed infections) but also underrepresented genetic variants, which would have been missed using Sanger sequencing. This study has expanded the number of subtypes recorded in free-ranging wild ruminants from the Iberian Peninsula and demonstrates the potential for spillover and spillback events between wild and domestic ruminant species sharing habitat. Similar ecologies of wildlife and livestock species create overlapping interfaces with the potential for *Blastocystis* transmission. Thus, it is crucial to carry out more studies evaluating the presence of this protist in other wildlife species with and without contact with livestock to improve our knowledge of its epidemiology and the risks it poses to livestock and human health.

## Supplementary Information

**Additional file 1.** **Summary of the sampling sites in Portugal according to bioregion with an emphasis on environmental, wildlife and flora features, adapted from references PNVSFS **[39]** and **[40]**.** The total number of wild ruminant faecal samples collected in each location is indicated.

**Additional file 2.** **Full dataset showing the epidemiological data used in the analyses conducted in this study, as well as the diagnostic and molecular results obtained.**

**Additional file 3.** **Distribution of *Blastocystis* subtypes/subtype subgroups according to free-ranging wild ruminant species and country of origin.** The asterisk denotes a potential novel ST10 subgroup based on sequence similarity and phylogenetic clustering. Coloured subtypes were detected in a single host species: ST10* in red deer, ST49 in roe deer, and ST26, ST30, and ST42b in fallow deer.

**Additional file 4.** **Percentage of *Blastocystis* subtypes and unique genetic variants observed by using next-generation amplicon sequencing among the *Blastocystis*-positive free-ranging wild ruminants from Spain and Portugal**.

**Additional file 5.** **Relative abundance of each *Blastocystis* ST detected in Spanish and Portuguese red deer according to Bioregion of origin.** A colour was attributed to each ST or ST subgroup identified.

**Additional file 6.** **Relative abundance of each *Blastocystis* ST detected in Spanish and Portuguese roe deer according to Bioregion of origin**. A colour was attributed to each ST or ST subgroup identified.

**Additional file 7.** **Relative abundance of each *Blastocystis* ST detected in Spanish and Portuguese ruminants according to Bioregion of origin.** From left to right species icons correspond to fallow deer, Iberian ibex, Southern chamois, and Barbary sheep. A colour was attributed to each ST or ST subgroup identified.

## Data Availability

The data that support the findings of this study are available within the main body of the manuscript and its supplementary material.
