# Supplementary material for: Blastocystis in free-ranging wild ruminant species across the Iberian Peninsula
Source: Vet Res. 2025 Jul 9;56:145. doi: 10.1186/s13567-025-01563-3 (PMC12239363; doi:10.1186/s13567-025-01563-3)
Supplement: Supplementary file 1 — Additional file 1. Summary of the sampling sites in Portugal according to bioregion with an emphasis on environmental, wildlife and flora features, adapted from references PNVSFS [39] and [40]. The total number of wild ruminant faecal samples collected in each location is indicated. [file 13567_2025_1563_MOESM1_ESM.docx]

**Additional file 1.** **Summary of the sampling sites in Portugal according to bioregion with an emphasis on environmental, wildlife and flora features, adapted from references PNVSFS [39] and [40].** The total number of wild ruminant faecal samples collected in each location is indicated.

| **Location** | **Bioregion** | **Environmental features** | **Wildlife and domestic animal’s^1^ features** | **Sampling site features** |
| --- | --- | --- | --- | --- |
| Montesinho Natural Park (MNP) | BR2 | Continental Mediterranean climate. Dry, hot summers, dry, cold winters. Open, cereal landscapes with pine or oak woodlands, limited to the north by mountains | Red deer and roe deer widely distributed. Wild boar abundant. Cattle *n* = 4816; goat *n* = 1717; sheep *n* = 34773; pig *n =* 1153. | Samples collected: 56. Oak and chestnut forests (e.g., *Quercus pyrenaica*, *Castanea sativa*) and shrub vegetation (e.g., *Ulex europaeus*, *Cistus landanifer*). AR: 438–1,481; MAP: 1200; MAT: 9.0 |
| Lousã Mountains  (LM) | BR1 | Atlantic climate with high precipitation rates. Pastures and deciduous woodlands. Mountainous habitats | Red deer and roe deer widely distributed. Wild boar abundant. Cattle *n* = 1064; goat *n* = 175; sheep *n* = 2127; pig *n =* 1053. | Samples collected: 52. Coniferous forests (e.g., *Pinus sylvestris*, *Pinus pinaster*) and shrubs (e.g., *Ulex* spp., *Erica* spp., *Calluna vulgaris*). AR: 300-1200; MAP: 1071; MAT 13.6 |
| Central Portugal East (CPE) | BR3 | Continental thermo Mediterranean climate. Pastures and crops with interspersed vegetation, sometimes forming savannah-like structures. Low altitude mountains with scrubland | Roe deer widely distributed, and red deer with limited distribution. Wild boar abundant. Cattle *n =* 7700; goat *n* = 1643; sheep *n* = 19810; pig *n* = 6115. | Samples collected: 5. Scrublands (e.g., *Cistus* spp., *Juniperu*s spp.) and tree species (e.g., *Quercus. rotundifolia*, *Quercus pyrenaica*). AR: 700–1,000. MAP: 1,195; MAT: 10.5 |
| Malcata Nature Reserve (MNR) | BR3 | Continental thermo Mediterranean climate. Pastures and crops with interspersed vegetation, sometimes forming savannah-like structures. Low altitude mountains with scrubland | Roe deer, widely distributed, and red deer with limited distribution. Wild boar abundant. Cattle *n =* 7663; goat *n* = 2873; sheep *n* = 63736; pig *n =* 3208. | Samples collected: 21. Scrublands (e.g., *Cistus* spp., *Erica* spp.) and tree species (e.g., *Pinus pinaster*, *Quercus suber*). AR: 425–1,078; MAP: 849; MAT 15.7 |

**^1^** Number of livestock animals across the locations (MNP, LM, CPE and MNR). Numbers corresponding to the 2017 data provided by the Portuguese Direção Geral de Alimentação e Veterinária (DGAV). Insufficient data to determine which animals are raised under intensive and extensive systems.

AR, Altitude range, in metres; MAP, Mean annual precipitations, in millimetres; MAT, Mean annual temperature, in Celsius degrees.
