# Supplementary material for: Blastocystis in free-ranging wild ruminant species across the Iberian Peninsula
Source: Vet Res. 2025 Jul 9;56:145. doi: 10.1186/s13567-025-01563-3 (PMC12239363; doi:10.1186/s13567-025-01563-3)
Supplement: Supplementary file 4 — Additional file 4. Percentage of Blastocystis subtypes and unique genetic variants observed by using next-generation amplicon sequencing among the Blastocystis-positive free-ranging wild ruminants from Spain and Portugal. [file 13567_2025_1563_MOESM4_ESM.docx]

**Additional file 4. Percentage of *Blastocystis* subtypes and unique genetic variants observed by using next-generation amplicon sequencing (NGS) among the *Blastocystis*-positive free-ranging wild ruminants from Spain (*n* = 30) and Portugal (*n* = 44).**

| **Subtype** | **Subgroup** | **No. of samples** | **No. of unique genetic variants** | **Percentage of positive samples** | **GenBank accession number(s)^b^** |
| --- | --- | --- | --- | --- | --- |
| ST2 |  | 5 | 3 | 4.3 | PV069303, PV069304, PV069313, PV069315 |
| ST5 |  | 9 | 4 | 7.8 | PV069309, PV069310, PV069311, PV069312, PV069314, PV069316 |
| ST10 | ST10a | 10 | 2 | 1.7 | PV069249, PV069250, PV069251, PV069301 |
|  | ST10b | 20 | 5 | 17.4 | PV069255, PV069256, PV069257, PV069258, PV069259, PV069260, PV069261, PV069262, PV069263, PV069264, PV069273 |
|  | ST10**^a^** | 3 | 1 | 2.6 | PV069271 |
| ST13 |  | 28 | 1 | 24.3 | PV069227, PV069228, PV069229, PV069230 |
| ST14 |  | 8 | 5 | 7.0 | PV069265, PV069266, PV069274, PV069282, PV069305, PV069308 |
| ST21 |  | 11 | 4 | 9.6 | PV069267, PV069268, PV069269, PV069277, PV069283, PV069284, PV069288 |
| ST23 |  | 4 | 1 | 3.5 | PV069292, PV069293 |
| ST24 | ST24a | 42 | 6 | 36.5 | PV069240, PV069241, PV069242, PV069243, V069244, PV069245, PV069246, PV069253, PV069254, PV069270, PV069275, PV069276 |
|  | ST24b | 59 | 7 | 51.3 | PV069231, PV069232, PV069233, PV069234, PV069235, PV069237, PV069238, PV069239, PV069252, PV069272, PV069294, PV069302, PV069307 |
|  | ST24c | 21 | 3 | 18.3 | PV069236, PV069278, PV069279, PV069280, PV069281, PV069287 |
| ST25 |  | 2 | 1 | 1.7 | PV069285, PV069286 |
| ST26 |  | 1 | 1 | 0.9 | PV069300 |
| ST30 |  | 1 | 1 | 0.9 | PV069306 |
| ST42b |  | 2 | 1 | 1.7 | PV069299 |
| ST43 |  | 2 | 1 | 1.7 | PV069295, PV069296 |
| ST44 |  | 6 | 3 | 5.2 | PV069289, PV069290, PV069291, PV069297, PV069298, PV069317 |
| ST49 |  | 5 | 1 | 4.3 | PV069247, PV069248 |

^a^ Potential novel ST10 subgroup based on sequence similarity and phylogenetic clustering.

^b^ Note that number of GenBank Accession numbers do not correspond in all cases to the number of unique variants as some genetic variants were present in more than one host or country and have more than one GenBank Accession number.
